# Biocompatibility pathways and mechanisms for bioactive materials: *The bioactivity zone*

**DOI:** 10.1016/j.bioactmat.2021.08.014

**Published:** 2021-08-26

**Authors:** David F. Williams

**Affiliations:** Wake Forest Institute of Regenerative Medicine, 391 Technology Way. Winston-Salem, North Carolina, 27101, USA

**Keywords:** Bioactivity, Host response, Biomaterial, Medical technology, Cell signaling

## Abstract

This essay analyzes the scientific evidence that forms the basis of bioactive materials, covering the fundamental understanding of bioactivity phenomena and correlation with the mechanisms of biocompatibility of biomaterials. This is a detailed assessment of performance in areas such as bone-induction, cell adhesion, immunomodulation, thrombogenicity and antimicrobial behavior. Bioactivity is the modulation of biological activity by characteristics of the interfacial region that incorporates the material surface and the immediate local host tissue. Although the term ‘bioactive material’ is widely used and has a well understood general meaning, it would be useful now to concentrate on this interfacial region, considered as ‘*the bioactivity zone’*. Bioactivity phenomena are either due to topographical/micromechanical characteristics, or to biologically active species that are presented in the bioactivity zone. Examples of topographical/micromechanical effects are the modulation of the osteoblast – osteoclast balance, nanotopographical regulation of cell adhesion, and bactericidal nanostructures. Regulation of bioactivity by biologically active species include their influence, especially of metal ions, on signaling pathways in bone formation, the role of cell adhesion molecules and bioactive peptides in cell attachment, macrophage polarization by immunoregulatory molecules and antimicrobial peptides. While much experimental data exists to demonstrate the potential of such phenomena, there are considerable barriers to their effective clinical translation. This essay shows that there is solid scientific evidence of the existence of bioactivity mechanisms that are associated with some types of biomaterials, especially when the material is modified in a manner designed to specifically induce that activity.

## 1 Introduction

This Leading Opinion Paper is an essay about the relationships between bioactivity and biocompatibility. The opinions that I express here are arranged in a logical sequence, as follows:

• Bioactivity can be defined as the effect of a substance upon a living organism or on living tissue. This is a very generic definition and does not lead to any practical consequences.
• On the other hand, a bioactive material can be considered as any material which has been designed to induce specific biological activity, or, of more relevance to medical technology, “*a biomaterial that is designed to elicit or modulate biological activity*” [1].
• In this context, a biomaterial is “*a material designed to take a form that can direct, through interactions with living systems, the course of any therapeutic or diagnostic procedure*” [2].
• From engineering and regulatory perspectives, the ‘form’ that the biomaterials take is referred to as a device [3]; this needs to have appropriate qualities of biocompatibility and functionality.
• Biocompatibility has, for 40 years, been defined as “*the ability of a material to perform with an appropriate host response in a specific application*” [2].
• It follows that any bioactive material that is intended to be used in a medical technology application should “**beneficially and appropriately direct interactions between the device and the host system through the modulation of biological activity**”.
• This process should be intentional (i.e., by design and not serendipity), which implies that the interactions with the host are well understood; in the context of the totality of biocompatibility phenomena, there should be satisfactory empirical evidence of the biocompatibility pathways that are involved [4].
• To date, there is little evidence of an understanding of these pathways. The translation of the theoretical aspects of bioactive materials to clinical applications would be significantly enhanced by embracing this concept of biocompatibility pathways for these materials and devices.

Although I often dwell on the correct meaning of words, this essay is not about semantics but on the real meaning of words in biomaterials science that are, so often, misunderstood, or indeed misused.

## 2 Concepts of inertness and activity in medical devices

In the early years of the clinical applications of biomaterials and implantable medical devices, there was only a poor understanding of biocompatibility. More or less by default, the host response came to be considered as a combination of a perturbation of classical wound healing and the degree of ‘biomaterial toxicity’ that, whatever precise mechanism was involved, influenced the host, both systemically and locally. The initial euphoria of being able to place a wide variety of materials, including metals and alloys, glasses and ceramics, natural and synthetic polymers and composites, into the body for the treatment of patients, with at least some success, gradually subsided as it was realized that this material selection process should be somewhat more refined. Thus, readily available alloys such as vanadium steel and bronze gave way to stainless steel, cobalt alloys and titanium alloys. Engineering plastics such as nylon and polyacetals were replaced by polyolefins, fluorocarbon polymers, acrylics and some silicone elastomers. Whatever the class of material, this selection process was guided by the over-riding need for the material to be as ‘inert’ as possible [5]. This was a contentious issue since it could be argued, with some scientific sense, that no material is fully inert within the body; there always has to be some interaction, especially at the interface. However, the concept of biomaterial inertness was translated into the consequences of the biocompatibility definition, since the response had to be ‘appropriate’ and appropriateness has to relate to the lack of clinically relevant adverse effects within the host. Thus, with alloys, the asymptotic curve of appropriate inertness was associated with the improvement of corrosion resistance through minimizing the release of metal ions or particulate products. With polymers, the improved inertness came with molecular design to minimize water absorption, hydrolysis, oxidation and the release of additives and contaminants.

The starting point for clinical success with many implantable devices, and also with extracorporeal systems, is inertness-controlled biocompatibility. This, of course, is not the only relevant factor, and processes of mechanobiology, especially the effects of mechanotransduction arising from the interplay of forces between biomaterial and tissues, must be considered, as have the idiosyncratic patient-derived effects and the consequences of clinical skill variables. Inertness, along with functionality, controls the choice of biomaterials for joint prostheses, cardiovascular devices, dental and maxillofacial implants, surgical meshes, ophthalmological products and so on.

Even with optimally inert biomaterials, however, the functionality is limited. The products mentioned in the last paragraph are all associated with the replacement or augmentation of mechanical or physical functions of certain tissues and organs. It has not usually been the intention that they replace or augment the specific biological functionality of these tissues. Traditional inert devices, however clinically successful they may be, do not actively and intentionally interact at a biological level with tissue systems. This situation is unlikely to be tenable within medical technologies other than traditional engineering replacements, such as tissue engineering and drug/gene/contrast agent delivery.

This is where bioactive materials come in, for, as noted above, they are designed to elicit or modulate biological activity. This is not a trivial issue, and we have to be very clear what we mean by ‘elicit’, ‘modulate’ and ‘biological activity’. One of our difficulties is that the concept of bioactive materials has been around for decades but the practicalities of bioactivity in relation to medical technology have developed spontaneously, even haphazardly, so there is little connectivity between the mechanisms that are involved. To emphasize this point, consider the roles that two of the most successful inert biomaterials play in orthopedic and spinal surgery. These are titanium (either the pure metal or one of a select series of alloys) and PEEK, polyetheretherketone. Their uses are dependent on the combination of inertness and mechanical properties. They are not bioactive materials. Recently, papers have been published concerned with ‘improving their bioactivity’ [6,7]. A central thesis of this essay is that biomaterials are designed to be either inert or bioactive; no material can be both at the same time. I will return to this point later.

The next crucial point is that the biological activity must be elicited or modulated, both of which terms explicitly meaning that there is a significant degree of control exerted by the material; the response is neither passive nor accidental. If we consider an inert surface such as the pure titanium mentioned above, it will have a measurable surface energy. This is a property or characteristic based on the laws of physical chemistry and metallurgy, certainly not of biology. If that surface is treated by some chemical or physical process (e.g., etching or machining), the surface energy may change a little. Under *in vitro* conditions, cells may show slight differences in their behavior (e.g., adhesion) on these marginally different surfaces. The question then arises as to whether that modified surface is now bioactive. Common sense might suggest that the entirely non-biological change on the material surface cannot be considered part of a bioactivity phenomenon. This essay will try to produce some resolution to this conundrum.

## 3 Categories of bioactive material

Because of the variable characteristics of bioactivity, and indeed the expansiveness of the requirement to ‘modulate biological activity’, there is no easy way in which bioactive materials could be classified, and nothing would be gained from any nebulous classification. It is possible, however, to identify certain mechanisms by which bioactivity is exercised, which should lead to the characterization of bioactivity pathways.

As with the pathways of biocompatibility [4], the bioactivity pathways are not entirely new biological phenomena, but biological processes that normally contribute to the performance of living systems, and which can be identified as being, at least partly, responsible for a biomaterial's performance. In each situation where bioactive materials may have beneficial clinical outcomes, the most appropriate way to identify putative pathways for that activity would be to start with the fundamental mechanisms associated with the inherent biological characteristics of the target tissues and/or cells and then identify any way in which the biomaterial could possibly influence those characteristics. This is surely better than the phenomenological process of observing some outcomes under experimental conditions and extrapolating to potential clinical outcomes.

Biomaterials – based processes that are potentially associated with bioactivity include (but are not limited to) the following:

o Materials that promote bone formation with or without hydroxyapatite deposition
o Materials that promote or inhibit cell adhesion in soft tissues
o Materials that promote endothelialization
o Materials that modulate inflammation
o Immunomodulatory materials
o Materials with antioxidant activity
o Materials that promote wound healing
o Anti-infective materials
o Anti-thrombogenic materials

Several of these phenomena are discussed below. It should be noted that this essay will not specifically address tissue engineering/regenerative medicine scenarios.

### 3.1 Materials that promote bone formation with or without hydroxyapatite deposition

This is the area that has received by far the most attention, both experimentally and clinically. Biomaterials, either monolithic or as surface coatings, that have been claimed to have bone-inducing or bone bonding properties include calcium phosphates such as hydroxyapatite [8], tricalcium phosphate [9], their biphasic forms [10] or polymer composites [11], bioactive glasses [12] including silicate, phosphate, borate and mixed glass systems [13], calcium carbonate – based materials such as coral or nacre [14], graphene or other nanostructured carbon derivatives and their composites [15], collagen – based materials [16], chitosan and other biopolymer hydrogels [17], bisphosphonate-functionalized materials [18], magnesium-based alloys [19], nanoscale modified titanium [20] and others.

The question arises as to what characteristics do these materials and/or coatings have in common that could accelerate or induce bone to form in their vicinity. It is of some interest to note that many of these putative bioactive materials are themselves often ‘enhanced’ by the inclusion of some agent that has its own bioactive characteristics, for example, silicon substituted bioactive glasses and calcium phosphates [21].

#### 3.1.1 Mechanisms of bone formation

If the bioactivity relates to the formation of new bone at or near the biomaterial surface, then it is important to discuss the performance of the material in the context of the essential biology of bone formation. It is emphasized that this activity concerns bone as a tissue, with all its complexity; it is insufficient for a material to solely induce hydroxyapatite deposition on its surface, since that is not functioning bone.

Bone is a mineralized connective tissue, the properties of which critically depend on its cellular components and the extracellular matrix (ECM), and the interactions between these [22]. Bone formation occurs during skeletal development and during repair, for example following injury [23]. Concentrating for the moment on the situation of a biomaterial placed within a site that has been prepared by surgical procedures, which is the case for the vast majority of applications within orthopedics, dentistry and maxillofacial reconstruction, the response to that biomaterial must be considered in the context of bone repair, alongside the normal host response to implanted devices. This host response involves mechanisms of inflammation and fibrosis [4,5]; the default position when a biomaterial is placed within a bone site is the formation of an interfacial zone of fibrous tissue. For bioactivity to become a reality, the bone repair process has to dominate fibrous repair.

Bone repair is an extreme variation within the dynamic environment of bone remodeling. Throughout life, there is a constant turnover of bone, as it is both resorbed and re-formed. Resorption takes place *via* the activity of osteoclast cells, while formation is caused by the activity of osteoblast cells. In a healthy adult, there is a turnover of about 10% of bone per year; in some metabolic diseases, such as osteoporosis, the balance is upset, with more resorption than formation. Not surprisingly, there is quite a large range in this remodeling process caused by genetic variation involving genes that encode regulators of bone homeostasis [24]. This could explain significant variations in clinical outcomes with bioactive surfaces, although too little is known about this for any significant conclusions to be drawn at this stage.

The critical cell in bone formation, and therefore in any bone bioactivity process, is the osteoblast. However, osteoblasts do not function in isolation. During bone remodeling, there is significant crosstalk between osteoblasts and osteoclasts [25], which allows bidirectional transduction of activation signals and the regulation and survival of both types of cells. Bone marrow derived macrophages also influence osteoblast activity through cytokine secretion [26]. Importantly, the ECM, which is produced by the osteoblasts, has an ongoing relationship with both osteoclasts and osteoblasts. The ECM components contribute a network of signaling mechanisms that influence bone metabolism and affect cellular processes such as proliferation, differentiation, and migration, regulating both osteoblast and osteoclast lineages [27]. Structurally, the ECM consists of collagen, largely Type I, some non-collagenous proteins, mostly glycoproteins such as alkaline phosphatase and sialoproteins, hydroxyapatite crystals, some lipids and water. This very brief summary of the osteoblast environment indicates significant complexity, which proposed bioactive materials must recapitulate.

Bone formation in normal remodeling involves a highly regulated cycle [28], with activation, resorption, reversal, formation, and termination phases. The process is orchestrated by osteocytes, which are long living cells, comprising 90–95% of cells in bone, that are formed when osteoblasts are buried in the mineralized matrix of bone; they form dendritic processes within the canaliculi, providing networks that interface with other osteocytes and other cells, sensing the local and systemic environment within the bone. Local regulation is critical to this sequence, although this is supported by systemic released factors.

#### 3.1.2 Signaling pathways in bone formation and effects of bioactive materials

Two signaling pathways dominate this regulation, the RANKL/RANK/OPG and Wnt pathways, although several others are involved. Mechanisms of Wnt signaling and cellular metabolism in bone, and specifically in relation to osteoblasts, have recently been analyzed by Karner and Long [29] and by Grigorie and Lerner [30]. Wnt genes comprise a family of signaling molecules that influence a wide range of developmental and disease processes [31]. This family was denoted Wnt, short for Int1/Wingless because first discoveries were of their role in tumorigenic growth (hence Int1, the first common integration site) and in the developmental processes in the fruit fly (where a gene mutation led to the formation of flies without wings). Wnt proteins transit across cells through the secretory pathway, associating with several proteins in the endoplasmic reticulum and the Golgi apparatus, with prominent glycosylation and acetylation modifications. Once in the extracellular milieu, they act as ligands, binding to co-receptors of the LRP family (low density lipoprotein receptor-related proteins) and to the signaling receptors Frizzleds, which belong to the family of G-proteins. When the heterotrimeric Wnt-LRP-Frizzled complex is activated by Wnt binding, Frizzled signaling takes place in the cell cytosol, resulting in activation of the protein β-catenin, which translocates to the nucleus, and which may regulate many cell – cell adhesion and gene expression processes.

Genetic studies have shown a causal relationship between Wnt signaling and bone formation. In experimental animals, manipulation of genes can alter bone mass and cause either osteoporosis or osteosclerosis. β-catenin is clearly involved in the mediation of Wnt signaling, acting at multiple stages of osteoblast differentiation and the regulation of both osteoblasts and osteoclasts. It is of considerable interest to identify whether bioactive materials with putative bone forming ability are able to influence this Wnt pathway. There have been several suggestions that this happens. Osteoblast precursor cells, MC3T3-EI, were shown to have their osteogenic potential upregulated *via* the Wnt- β-catenin pathway *in vitro* on nanostructured titanium surfaces in comparison with machined titanium controls [32]. Liu et al. reported that lithium-doped titanium surfaces were associated with osteogenic differentiation by activation of the Wnt- β-catenin pathway [33]. Bolander et al. provided *in vivo* evidence that calcium phosphate biomaterials enhanced bone formation through activation of the Wnt pathway, along with BMP and PKC pathways [34]. Abaricia et al. investigated the possibility that Wnt signaling and macrophage activation are inter-related during the inflammatory response to intramedullary titanium rods [35].

The role of substrate-dependent Wnt signaling with mesenchymal stem cells (MSCs) has also be extensively investigated. Hsu and Huang cultured MSCs on chitosan and hyaluronan membranes and 3D spheroids and showed that Wnt signaling of these cells is substrate dependent under their *in vitro* conditions [36]. Go et al. identified the Wnt- β-catenin pathway as a major factor in the interactions between biomaterials and stem cells but were unable to specifically state how this pathway leads to greater or faster bone formation under practical conditions [37].

The RANKL/RANK/OPG system is extremely important in bone physiology [38] and is the basis for several clinical therapies for osteoporosis and bone cancers [39]. Cytokines and hormones that promote osteoclast formation first act on cells of the osteoblast lineage, promoting the production of the regulator of osteoclastogenesis. This regulator was found to be the receptor activator of the NF-kB ligand, known as RANKL. This acts upon its receptor RANK in the hematopoietic lineage, which is restricted by a decoy soluble receptor osteoprotegerin, OPG, a product of osteoblasts. There is, therefore a dual RANKL-RANK signaling interaction that influences both osteoclast and osteoblast functions.

**Fig. 1:**
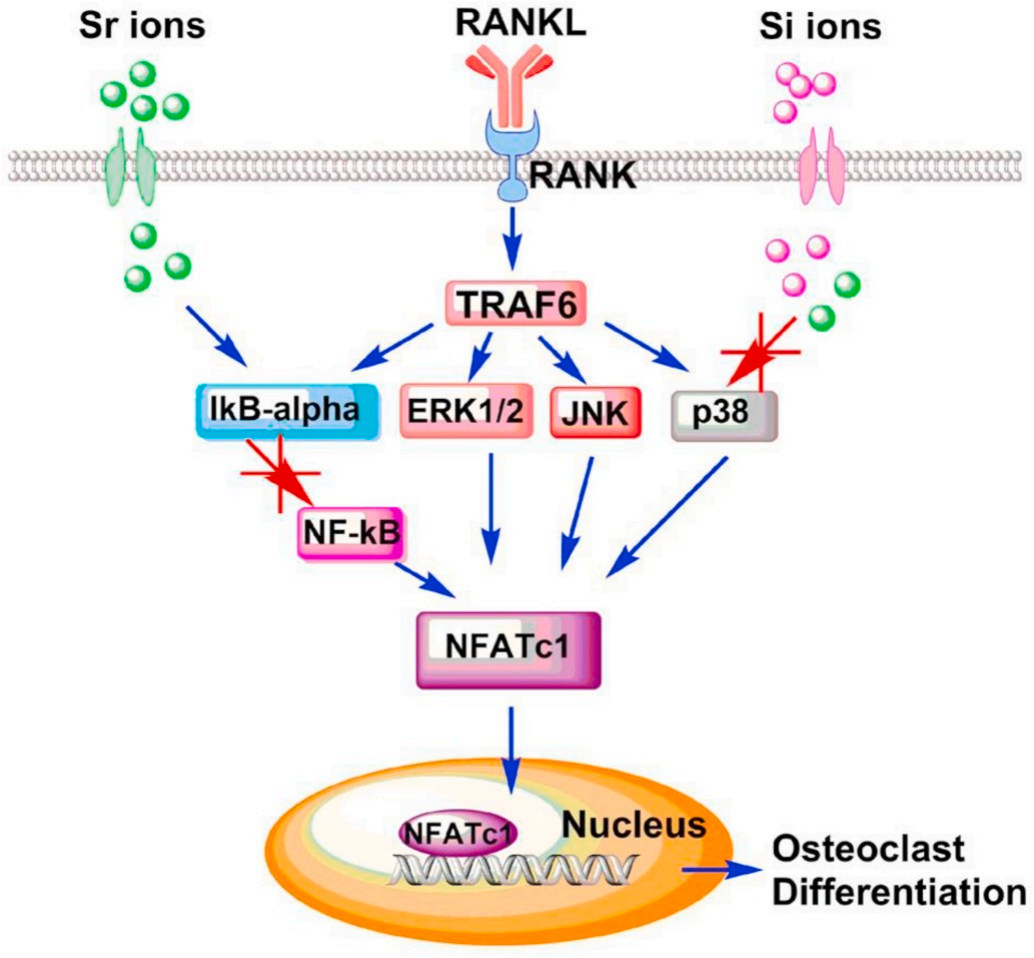
Proposed mechanism of inhibited RANKL-induced osteoclastogenic differentiation of Sr-SBG through blocking NF-κB signaling pathway and p38 signaling pathway which would result in decreased translocation of NFATc1 and eventually inhibit osteoclastogenesis. Reproduced from Huang et al., [40] Regen. Biomater. 7 (2020) 303–311, with permission.

Again, there have been suggestions that bioactive materials could influence this RANKL/RANK/OPG system, especially by inhibiting osteoclastogenesis, promoting bone formation rather than resorption. Several of these suggestions concern the role of biomaterial-derived cations. Huang et al. indicated that strontium-substituted bioactive glasses inhibit osteoclastogenesis through suppression of the RANKL-induced signaling pathway [40] as shown in Fig. 1. On the basis of *in vitro* evidence with RAW 264.7 macrophages, it was suggested that the Sr ion had a strong inhibitory effect on osteoclast differentiation by disruption of the RANLK-activated p38 signaling and NF-kB pathways. It is also believed that lithium ions released from the Li_2_Ca_2_Si_2_O_7_ phase of some bioactive glasses inhibits RANKL-induced osteoclastogenesis by macrophages *in vitro* [41].

The Hedgehog pathway (HH) is also a potentially relevant signal transduction pathway, or more correctly, a series of related pathways. In the canonical pathway, HH signaling is initiated by one of three ligands, Sonic Hedgehog (SHH), Indian Hedgehog (IHH) and Desert Hedgehog (DHH), each of which have distinct spatial and temporal expression patterns [42]. Their influence on bone development is well established [43]; SHH is a major morphogen in patterning limb buds and IHH is involved in endochondral ossification and induction of osteoblasts in the periosteum [43]. Zhang et al. have provided some preliminary *in vitro* evidence that bioactive glass ceramics can influence HH pathways [44]. Lin et al. suggest that micro/nanotextured surfaces affect proliferation and differentiation of MG63 osteosarcoma cells *in vitro via* the HH pathways [45]. One hint about the therapeutic potential to influence bone repair by enhancing HH signaling has been provided by Lee et al. using biomaterials that incorporate osteoinductive oxysterol liposomes, known to promote calvarial bone healing *in vivo* [46].

These are not the only relevant pathways, and there are many other extracellular and intracellular messenger molecules. Hsu and Huang [37] list around 30 such molecules, which, apart from those mentioned in the three pathways above, include IL-1, TNFα, TGFβ, BMP2/4/7, VEGF, MAPKs, Runx2 and Osterix. They also point out that osteoblasts, adipose tissue-derived stromal cells, MSCs and macrophages may be involved in these pathways. A detailed description of the genetic and transcriptional control of bone formation was published by Javed et al. [47]. There are many factors that are associated with bone formation, many of which are multifunctional and can cross-react with each other.

Knowledge about these pathways and messenger molecules, which is extensive but far from complete, should lead to the development of therapies and techniques to either promote or inhibit bone formation in certain clinical settings. This essay is only concerned about the potential procedures to promote bone formation. The most widely attempted approach involves drug-assisted bone healing, using, for example, growth factors, bisphosphonates, glucocorticoids, non-steroidal anti-inflammatory drugs, prostaglandins, enzyme inhibitors, statins and divalent metal ions, often with biomaterial-based delivery platforms [48]; there remains, however, a significant gap between preclinical discovery and clinical translation. A prime example of this dichotomy is seen with the TGFβ superfamily of growth factors, including BMPs and TGFβ1, which can regulate Wnt pathways but have wide-ranging effects on other biological processes that have troubling clinical side effects [49]. This is also seen with osterix, a transcription factor for osteoblast differentiation, where molecules such as parathyroid hormone can increase osterix expression levels, but can also lead to osteosarcoma development [50]. In some situations, the potential advantage of using gene transfer instead of direct growth factor delivery, for example with BMP-2 genes [51] has been considered, but safety concerns and high manufacturing costs have held up clinical translation.

When considering how putative bioactive materials can influence any of these bone formation pathways in clinical practice, it is clear that, at this stage, the direct chemical interaction between the biomaterial and any of the signaling molecules (either those mentioned above or others) is, with one exception discussed below, highly unlikely. This is not only due to manufacturing constraints but also to the fact that any such interactions would necessarily involve released active agents, which would place the product into a totally different regulatory category (i.e., combination products). Even more importantly, the specific targeting of any released molecules to one, and only one, part of a complex signaling pathway, is very challenging, and various possibilities exist for side effects.

The one exception involves metal ions, it being reported above that both strontium and lithium can, at least under *in vitro* conditions, influence some pathways. As discussed by Zhou et al., several other ions are potentially involved, especially with the regulation of stem cells [52]. Experiments have shown that Mn, Ca, Zn, Sr, Si, Cu, Co, Li and B ions all have some effect on regulating cell function and their release from biomaterials can, in theory, influence subsequent bone formation. Several of the pathways and cell types described earlier may be associated with these ions. The main difficulty in interpreting this behavior is that most biomaterials, especially bioactive glasses, release multiple types of ions, at different rates, some of which may have cytotoxic effects above certain levels, so that evidence of causation between biomaterials-released ions and bone bioactivity and their safety, is, at best, phenomenological and qualitative.

**Fig. 2:**
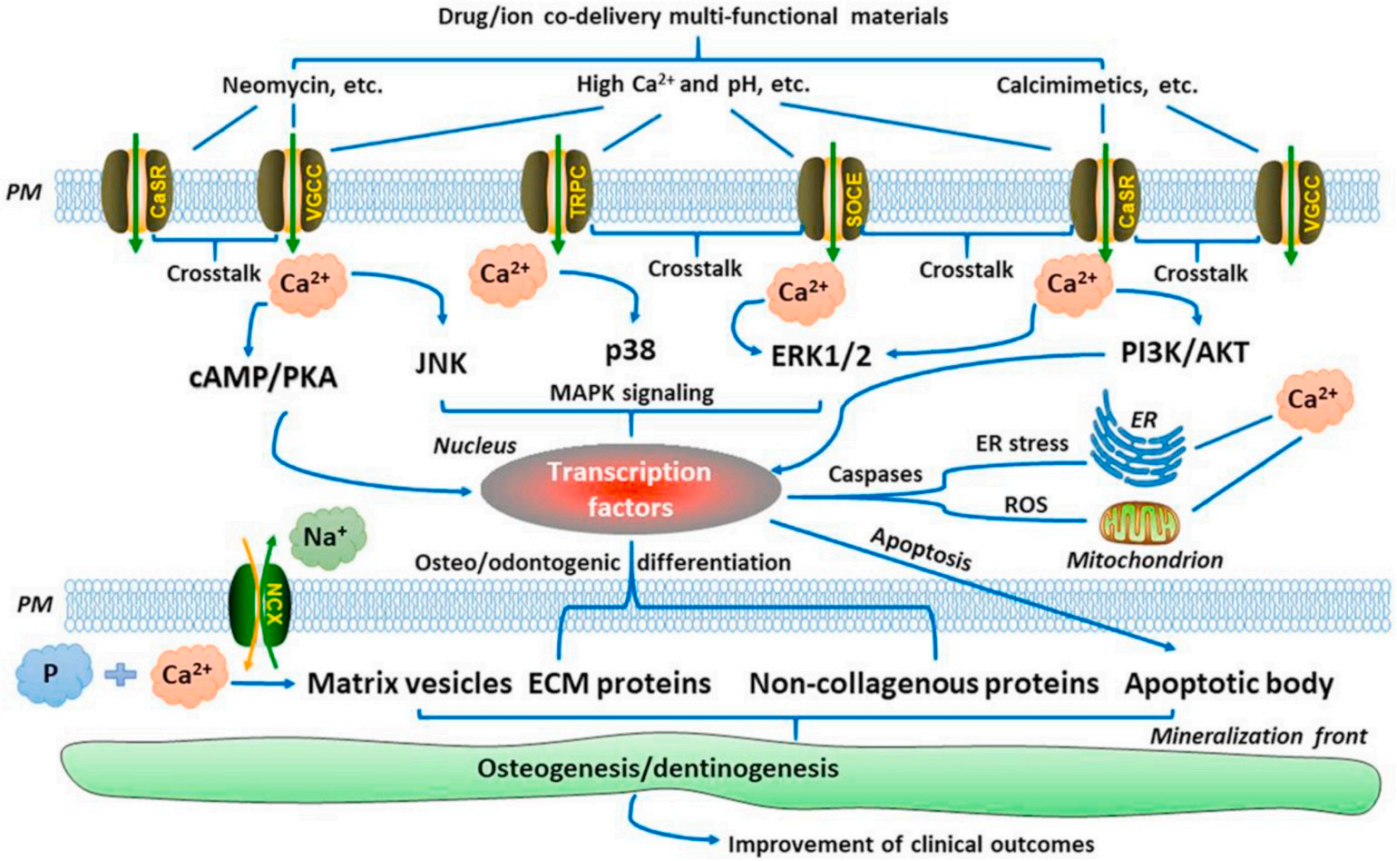
Ca^2+^ signaling as a tool in clinical treatment. Ca^2+^ released from scaffolds containing intracellular Ca^2+^ stores can modulate osteo/odontogenic differentiation and cell-mediated mineralization by activating Ca^2+^ channels–transporters and their downstream signaling pathways, such as the MAPK (ERK1/2, JNK, and p38), cAMP–PKA, and PI3K–AKT pathways. AKT: protein kinase B; cAMP: cyclic adenosine monophosphate; ECM: extracellular matrix; ER: endoplasmic reticulum; ERK1/2: extracellular signal-regulated kinase 1/2; JNK: c-Jun N-terminal kinase; MAPK: mitogen-activated protein kinase; P: inorganic phosphate; PI3K: phosphoinositide 3-kinase; PKA: protein kinase A; PM: plasma membrane; ROS: reactive oxygen species. Reproduced from An [53], J. Cell Physiol. 234 (2019) 2169–2193, with permission.

The role of calcium ions derived from calcium phosphate-based biomaterials is very relevant here; the simple fact that the mineral phase of bone is a calcium phosphate does not mean that all such biomaterials are, automatically, bioactive. As discussed by An [53], Ca^2+^ transport in the extracellular space is an important regulator of cell phenotype and the release of Ca^2+^ from materials or drugs could influence host responses, but this is a complex process which is dependent on ion concentrations in the tissue and the presence of downstream signaling molecules, shown in Fig. 2. It is possible that ion release from a biomaterial surface can activate Ca^2+^ channel transporters and thence one or more of the downstream signaling pathways, including the MAPK (ERK1/2, JNK and p38), cAMP-PKA and P13K-AKT pathways, resulting in activation of transcription factors in the nucleus and osteoblast differentiation. This sequence is dependent on many factors, especially those of kinetics, concentration, and equilibrium, so that bioactivity of calcium phosphate ceramics and related glasses is by no means guaranteed.

Since, apart from the release of metal ions, and in the absence of any classical pharmacological agents incorporated into a biomaterial, it is unlikely that bone-bioactive materials interact with these pathways through direct chemical interactions, it is necessary to consider alternative mechanisms. This inevitably points to mechanotransduction and, especially, to the role of surface topography in the distribution of mechanical forces at bone – biomaterial interfaces. The interplay between active biomaterials and mechanobiology was the focus of a recent essay by Ozkale et al. [54], where it was explained that cells are constantly interacting with their surroundings such that engagement with other cells and the ECM involves the formation of dynamic adhesions and the application of cellularly-generated forces by means of these adhesions. Through their interaction with both endogenous and exogenous forces that arise from a variety of factors, cells become involved with a variety of processes, including regeneration. It was noted that active biomaterials may be able to recapitulate the dynamic microenvironment within living tissues. They were mostly concerned with environmentally-responsive biomaterials (including those that respond to external physical fields) but the arguments also apply to the bioactive biomaterials that are the subject of the present paper. This position was further advanced with respect to porous biomaterials scaffolds and bone regeneration by Du et al.*,* who discussed the hierarchical design of materials and the influence of both internal cues and external stimuli [55].

**Fig. 3:**
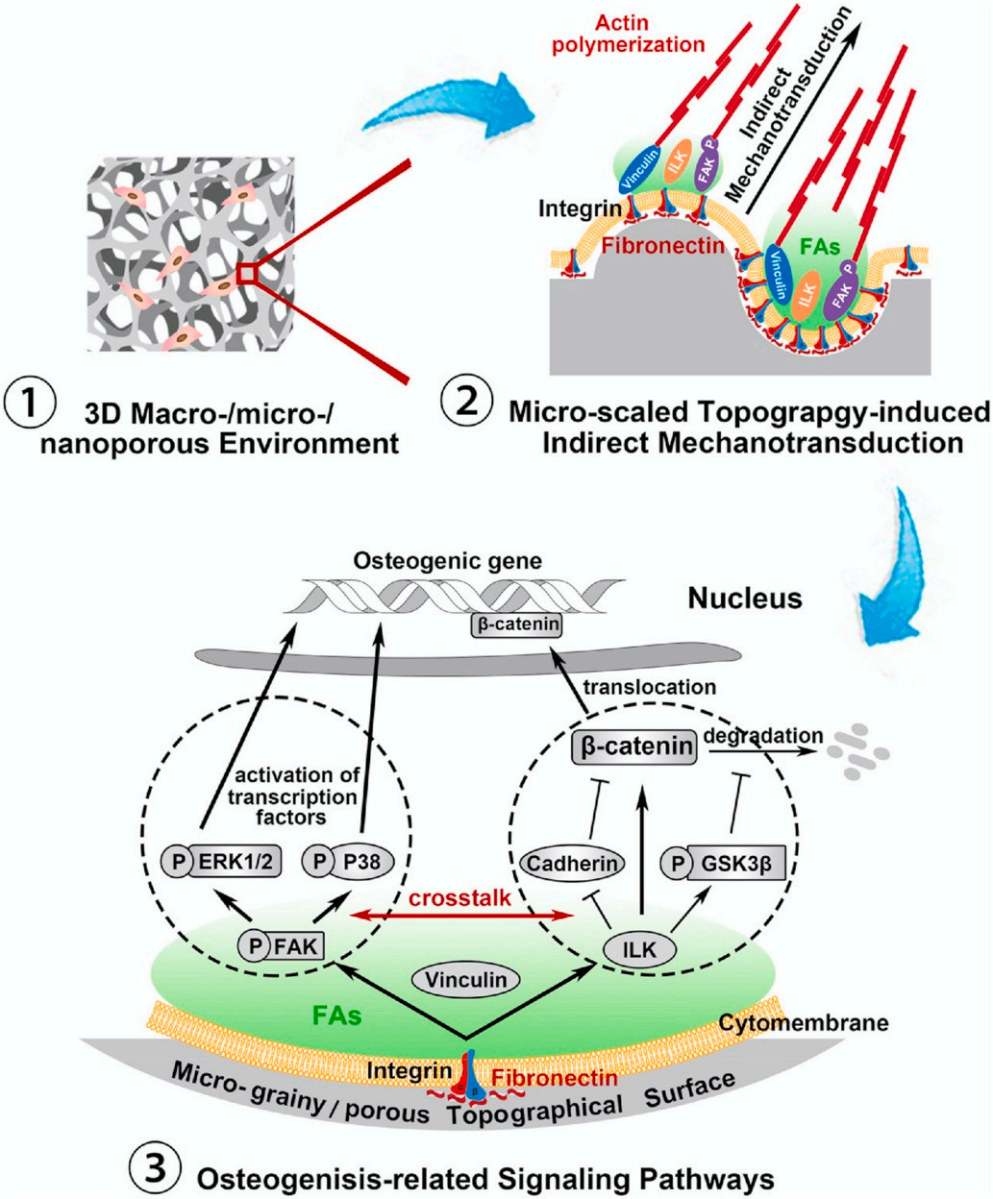
Schematic diagram of the microscaled topography-mediated osteogenic differentiation of MSCs. In a three-dimensional macro/nanoporous environment, the microscaled topography, especially the hybrid micrograiny/microporous topography, enhanced integrins and Fn expressions and thereby facilitated FAs assembly and actin filament polymerization. Via indirect mechanotransduction, FAK/MAPK and ILK/β-catenin signaling pathways and their crosstalk were activated, and consequently led to MSCs osteogenesis. Reproduced from Niu *et a*l, [56] ACS Biomater. Sci. Eng. 3 (2017) 3161–3175, with permission.

An extremely good example of how topographical cues can influence osteogenic differentiation pathways *via* mechanotransduction in the absence of chemical stimuli has been provided by Niu et al. [56]. This study investigated a series of microstructured topographies, involving an identical material in each, where osteogenic differentiation of MSCs varied with the nature of the topography. It was found that optimal mechanotransduction induced upstream expression of integrin subunits, focal adhesion complexes and, in consequence, the up-regulation of FAK/MAPK and ILK/β-catenin signaling cascades (Fig. 3). There is obviously considerable complexity within these mechanisms, as several other pathways may be involved [57]. The precise mechanisms by which stress (or strain) distribution within the topographical microenvironment, and their quantification, influence these pathways and cascades, are not known at this time, but the role of integrins and the integrin – focal adhesion complexes are likely to be important.

#### 3.1.3 Perspectives on bone-inducing bioactive materials

There is clearly no single bioactivity pathway for bone-inducing biomaterials. It is quite possible, or indeed probable, that a successful, functional, bone – biomaterial interface can be generated by minimizing or eradicating, an inflammatory response that leads to fibrosis at the interface. This is quite consistent with the classical concept of osseointegration but does not correspond to the requirements of a bioactive material as set out in this paper.

It would seem that there are two fundamental types of mechanism that could be involved with bone-inducing bioactive materials. The first is that of metal ion influence on downstream signaling pathways that control the balance between osteoblast and osteoclast behavior or the differentiation of stem cells into osteoblast or other lineages. There are several potential pathways and types of activating metal ion; calcium is likely to dominate but there are others.

The second is that of material topography – controlled mechanotransduction, where there is interplay between stresses at or near the interface and which are generated by endogenous or exogenous factors, determine how integrins and focal adhesions are able to manipulate the same type of pathway.

It is likely that these types of mechanisms could operate synergistically. This should not be surprising in view of the dominant control of biocompatibility phenomena in general by combinations of mechanotransduction and molecularly-induced physiological interactions [4].

### 3.2 Materials that promote or inhibit cell adhesion in soft tissues

Biocompatibility is profoundly influenced by the interactions between cells and material surfaces. Leaving aside those events concerned with bone discussed above, the majority of situations relate to the interactions of cells that reside in soft tissues. In some situations, it is important, indeed essential, for certain cells to physically come into contact with, and adhere to, the surface if the optimal host response is to be achieved. In other situations, it is preferable for certain cells to avoid attachment to surfaces. The control of cell behavior at biomaterial interfaces is, therefore, of extreme importance and bioactivity of these materials has been extensively discussed in this context.

A general review of the design of biomaterial surfaces intended to exert control over cell behavior has been recently published by Amani et al. [58]. They discuss the factors that may have such control, and which can be manipulated by surface modifications; they include surface chemistry, surface topography (including roughness and patterning), surface charge, wettability, surface energy and surface mechanical characteristics. Many of these are obviously inter-related; as the authors point out, individual mechanisms are often unclear, especially as *in vitro* quantitative data may be contradictory.

Joyce et al. have similarly reviewed material properties with respect to bioactivity but focused on natural biomaterials and the mechanisms involved [59]. Their discussion was also aimed at tissue engineering applications, but most points are relevant to the use of these biopolymers in a variety of medical technologies. The importance of structural elements, of the macromolecular sequences, of chain folding and linkages and overall architecture was emphasized since these factors directly control the cell – matrix interactions and subsequent responses. The cell surface receptors, including antibodies, lectin, CD44, E-cadherin, integrins, discoidin domain receptors 1/2, human osteoclast associated receptor and TLR toll like receptor may be able to recognize material motifs and activate specific signaling cascades. Some of these pathways, such as ERK 1/2 and NF-kB are directly associated with cell adhesion and can relate to motifs and receptors provided by macromolecules such as collagens and elastin.

As with bone bioactivity, there are two broad approaches to cell adhesiveness bioactivity. One is concerned with the use of adhesive peptides or equivalent cell-instructive molecules, the second with topography.

#### 3.2.1 Cell adhesive and cell instructive peptides

Peptides are molecules containing two or more amino acids in which the carboxyl group of one acid is linked to the amino group of another. Typically, there can be up to 50 amino acids in a peptide, larger arrangements being considered to be proteins; peptides that consist of less than 20 residues are generally considered to be ‘short peptides’. Peptides have a very diverse rage of biological properties which are utilized in many natural processes *in vivo*. Short peptides are usually easy to synthesize by chemical methods, or can be obtained recombinantly, and they are widely used in a variety of pharmaceutical and biotechnological applications [60]. The bioactivity characteristics of peptides range from cell adhesion (e.g., RGDs of fibronectin and YIGSR of laminin), to cell penetration (e.g., TAT peptide), antimicrobial effects (e.g., bacitracin), hormone activity (e.g., angiotensins), anticancer properties (e.g., bleomycins) and anti-oxidation (e.g., glutathione).

As reviewed by Collier and Segura ten years ago [61], these substances, especially the short synthetic peptides, have found increasing use as components of biomaterials within implantable devices and regenerative medicine over several decades, in order to exploit bioactivity. They are not without difficulties and disadvantages [62], particularly the fact that they can be degraded into fragments by proteolytic enzymes, and the general difficulty of persuading them to take up conformations that optimize their bioactivity [63]. Several solutions have been proposed to overcome these problems. Reese et al. discuss the development of what they refer to as peptidomimetics, which are sequence-controlled molecules that exhibit different folds and morphologies that create new structures and functions which mimic the natural peptides [63]. Alternatively, molecules may be synthesized with combinations of peptide sequences with complementary or synergistic effects so that they can address more than one biological target at the biomaterial surface [64]. Examples here include the ability to improve receptor selectivity by combining RGD with the PHSRN sequence and combining the RGD motif with BMP-2-derived peptides. In the former case, the non-specific binding of RGD with integrins is improved by incorporation of the PHSRN peptide of fibronectin, which increases the selectivity of RGD towards the α5β1 integrin. In the latter case, the combination of these two integrin binding ligands produces a more ECM-like microenvironment that could simultaneously enhance cell adhesion and cell differentiation.

With respect to the process of cell adhesion with bioactive materials, it should be noted that there are well-established mechanisms of cell-cell adhesion and cell-matrix adhesion; it is likely that cell-bioactive surface adhesion resembles these mechanisms. Crucial to the mechanisms of cell adhesion are the cell adhesion molecules (CAMs). These are proteins that are present in cell membranes, typically protruding from the membrane, which can recognize and attach to other molecules, usually CAMs themselves, on other cells or substrates. There are four main types of CAM (and a few minor examples), integrins, selectins, cadherins and members of the immunoglobulin superfamily, IgSF [65].

Integrins are large heterodimers consisting of α- and β-chains which bind to a variety of ligands in the ECM and on the surfaces of other cells [66]. The extracellular domain is large, containing several sub-domains, including the most important ligand binding domains. The transmembrane domains associate with each other at two motifs, which maintain the integrins in an inactive state. Their activity is regulated from within the cell through cell signaling initiated by other cell surface receptors. Of considerable importance are focal adhesions, specialized sites within cells where clustered integrin receptors interact with the ECM on the outside with the actin cytoskeleton on the inside of the cells, providing strong adhesion to the matrix and transmitting mechanical forces across the plasma membrane [67]. While integrin activity is dominant in the ECM, cadherins dominates cell – cell adhesion [68]. This is a superfamily of over 40 proteins. So-called classical cadherins, including E−, P-, N- and R-cadherins, are single membrane -spanning molecules with divergent extracellular domains of five repeats and a conserved cytoplasmic domain. There is only weak binding between the extracellular domains, but cell – cell adhesion is strong as it develops during lateral clustering that link the cytoplasmic domain to the actin cytoskeleton. Selectins are Ca^2+^ - dependent lectins that are important in cellular adhesion activity; these are the L- (expressed by leucocytes), P- (by platelets) and E− (by activated endothelial cells) selectins [69]. Selectins are particularly important in the cardiovascular space, where mediation of adhesion and signaling contribute to several diseases and conditions.

Knowledge of cell adhesion mechanisms, mediated *via* peptides or proteins, now has to be translated into meaningful systems that facilitate adhesion *in vivo* and in clinical settings. This has not been a straightforward process and few clear strategies have emerged with respect to cell-adhesive bioactive materials. Recent work on enhanced cellular behavior mediated by E-cadherin surface immobilization [70] and the regulation of cell behavior by synergistic integrin and growth factor signaling [71] show some promise, but the detailed observations are obtained during *in vitro* studies. Similarly, Barros et al. were able to demonstrate the potential of mono-PEGylated recombinant human N-terminal agrin as a natural affinity binding ligand for the site-selective immobilization of laminin and resulting mediation of cell adhesion and spreading [72].

One of the main difficulties in extrapolating from *in vitro* data on cell adhesion to clinically relevant applications of bioactive peptides relates to the dynamic nature of cell-material interactions *in vivo* [73]. As discussed by Pan et al.*,* cellular processes in human tissues are dependent on dynamic receptor-ligand interactions at the cell membrane – ECM interface [74]. Artificial matrices that have a reversible display of bioactive ligands could therefore be very important; the authors point to the possibility of using reversible covalent interactions or molecular assembly to dynamically immobilize cell adhesive peptides such as RGD on material surfaces to regulate cell adhesive behavior over time. Their own approach has involved an epitope-imprinting process where surface molecular recognition sites are used to bind an epitope-tagged bioactive peptide with an epitope tag at one terminus and a cell adhesive peptide at the other terminus. A similar approach has been presented by Clegg et al. [75].

#### 3.2.2 Cell adhesion and topography

As well as the chemically-based interactions discussed above, physical characteristics also play an important role in cell adhesion to substrates, both the natural ECM and biomaterials [76]. Two surface features have dominated arguments here, which essentially mirror the discussions about bone bioactivity in the earlier section; these are elasticity and topography, especially nanotopography. Again, many of the observations are derived from *in vitro* studies that involve two-dimensional interfaces; as such they are difficult to translate to realistic three-dimensional *in vivo* environments, a difficulty that is compounded by significant variations between cell types and the features of cell behavior. With respect to the ECM, a major factor is the ligand density related to any one cell type, where cell migration behavior is different between smooth muscle cells and endothelial cells, and between the ligand density on fibronectin and collagen [76]. There is far more evidence that nanotopography influences cell shape and morphology than it does adhesion.

**Fig. 4:**
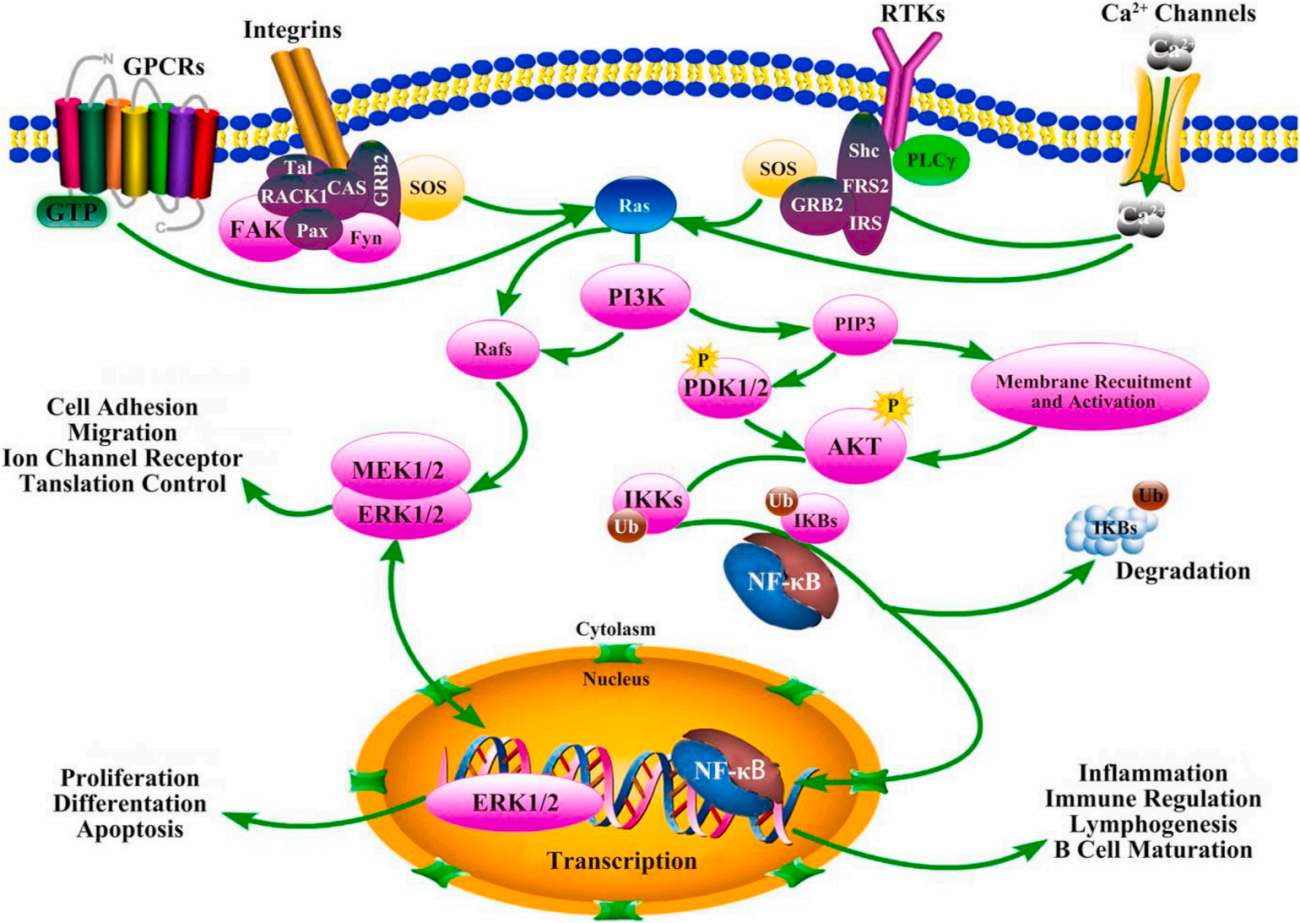
FAK–NF–κB signaling pathway mediates cell adhesion and the immune response of macrophages cultured on the surface of the nanostructure materials. Reproduced from Chen *et a*l, [77] Nano Lett. 19 (2019) 3480–3489, with permission.
• Material features that include the presence of biological cues, topography, and mechanical properties, combine to influence cytoskeletal crosstalk pathways in order to regulate cell adhesion [78].
• Topography at the micron scale rather than nanoscale can reduce cell adhesion with respect to macrophages and fibroblasts, through interference with cell proliferation during normal cell cycles [79].
• Microtopographical cues on synthetic nerve guides regulate mTOR gene expression (a central regulator of mammalian metabolism) during neurite outgrowth and glial migration during peripheral nerve regeneration [80].
• Nanotopography of fluorine-incorporated titania surfaces influences adhesion and differentiation of bone marrow derived stem cells through increased expression of extracellular signal regulated kinase (ERK) and cAMP response element-binding protein (CREB) and their combined ERK/CREB pathway [81].
• Lipid signaling on the actin cytoskeleton of fibroblasts is modulated by surface stiffness and microtopography *in vitro* through effects on phosphatidylinositol bisphosphonate (PIP2) and similar pathways [82].
• The nanotopography of amyloid fibrils incorporated into hydrogels can mediate active cellular adhesion *via* focal adhesions [83].

The observations on this matter are, not surprisingly, phenomenological rather than systematic, with the result that few, if any, definitive rules have emerged as how the various surface features can control cell adhesion. The following are some examples of these observations:

• Nanostructured titanium surfaces influence interfacial elasticity characteristics and can ‘regulate’ the immune response through control of the stretching of macrophages that are present in the interface region [77] (Fig. 4).

As discussed by Cimmino et al., [84], there is sufficient knowledge obtained under static conditions *in vitro* to demonstrate the potential mechanisms by which bioactive materials, programmed with specific surface features related to topography and biomechanics, should be able to control cell function, including cell adhesion. They argue that the development of platforms that display micro- and/or nano-scale dynamic signals, with physical-chemical stimuli necessary to actuate spatio-temporal changes in signaling patterns, should allow the translation of these mechanisms into clinical applications. The same view is held by Maynard et al. with respect to the fine-tuning of the spatial presentation of bioactive ligands within peptide-functionalized hydrogels [85].

#### 3.2.3 Perspectives on cell adhesion to biomaterials

As with bone-bonding biomaterials, there are two types of mechanisms by which bioactive materials could influence cell adhesions. Again, surface topography, and related aspects of mechanotransduction, provide one opportunity to influence cell adhesion. Secondly, cell adhesion molecules and especially cell-instructive peptides, presented by the material could enhance cell attachment and broader cell behavior.

### 3.3 Materials that promote endothelialization

The endothelium is the inner layer of the vascular wall and is composed of a monolayer of endothelial cells and a basal membrane consisting of Type IV collagen, laminin and some other ECM proteins. It constitutes the interface between blood and the rest of the vessel wall, maintaining homeostasis in the vascular system by regulating stimuli, both local and systemic. The functions of the intimal layer are controlled by wall shear stresses, circumferential stresses, and pulsatile pressure [86]. Mechanoreceptors on the surface of the endothelial cells sense these stresses and transduce them into intracellular signals, modulating cell morphology and function. The endothelial cells act as a protective barrier for the vessel wall, communicating with smooth muscle cells and fibroblasts to determine the response to injury or damage.

Any clinical intervention within the vascular system can produce some form of damage to the sensitive endothelium, initiating such responses. Ideally, for any implantable device placed within blood vessels, this response can influence both performance of the device and the health of the patient; in most circumstances, the generation of a new endothelium is considered the best response, which leads to the possibility that bioactive materials could be employed in order to facilitate this response.

Two principal implantable devices could be considered here, the vascular graft and the intravascular stent. However, this essay concentrates on the graft only; with stents, the situation is complicated by the use of drug elution methods, which have pharmacological activity [87] and by the use of biodegradable alloys, which operate by different mechanisms; these have been comprehensively reviewed in this journal very recently [88]. With respect to prosthetic vascular grafts, Zilla et al. discussed the difficulties of encouraging graft endothelialization a number of years ago [89]. In general, mechanisms of endothelialization can be divided into those which are attempted *in vitro* before implantation into patients and those which depend on processes *in situ*. *In vitro* endothelialization techniques, for example with fibrin pre-coating, can improve the patency of conventional polytetrafluoroethylene grafts [90], but the time required for the culturing process, typically 25 days, is a serious drawback for clinical application.

*In situ* endothelialization can occur by transanastomotic growth (where the host intima spreads from the anastomoses towards the graft center), by transmural capillarization (where endothelial cells pass through capillaries in the vessel wall) or by deposition of circulating cells from flowing blood [86]. The first two of these processes are slow and possibly self-limiting and are not readily amenable to control by bioactive materials. Most attempts to facilitate graft endothelialization have, therefore, focused on attracting deposition of cells from the circulating blood.

The status of technologies to improve homing of endothelial progenitor cells (EPCs) onto vascular grafts a decade ago was reviewed by Avci-Adali et al. [91]. There are relatively few of these cells in circulating blood, and their capture and differentiation into endothelial cells to create an autologous endothelium is not a trivial matter. The first strategy concerns the use of antibodies; EPCs have three surface markers, two of which, CD34 and VEGFR-2, can be mobilized onto polymeric vascular grafts to induce cell capture. Experimental observations with grafts and also clinical experience with stents showed that endothelialization was possible, but control of the responses of other cells, including smooth muscle cells, was problematic. Other strategies identified by these authors used ECM-derived peptides, oligosaccharides and aptamers, but all of these remained potential technologies rather than clinically-proven at that time.

**Fig. 5:**
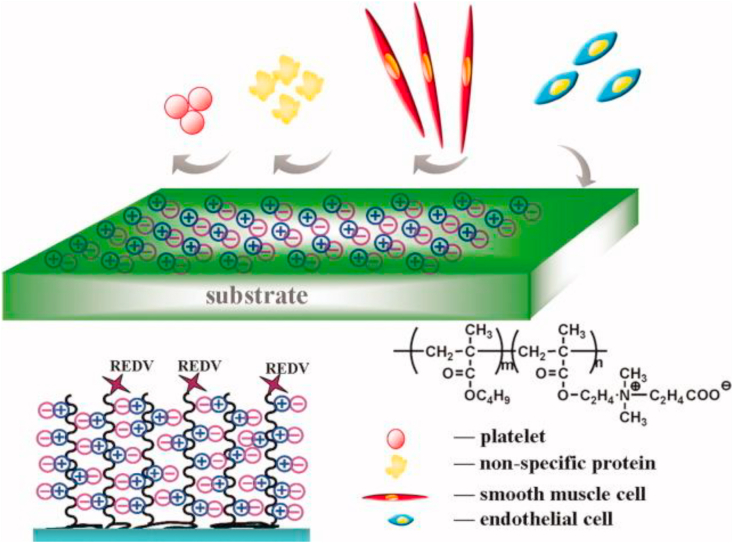
Random copolymers of carboxybetaine methacrylate (CBMA) and butyl methacrylate (BMA) were synthesized as coating materials, and REDV peptide was covalently conjugated onto polymer coating surface. The adhesion, proliferation, and migration of endothelial cells and smooth muscle cells were monitored, Reproduced from Ji et al.*,* [93] J. Biomed. Mater. Res. 100A (2012) 1387–1397, with permission.

In general, peptides are still the most commonly used substances to encourage endothelialization. Jun and West modified polyurethane grafts with polyethylene glycol combined with the cell adhesive peptide YIGSR [92]. Ji et al. used a zwitterionic polycarboxybetaine coating that was functionalized with REDV peptide, shown in Fig. 5 [93]. Lee et al. investigated polydopamine-mediated immobilization of an RGD – containing peptide together with basic fibroblast growth factor [94]. Both Peng et al. [95] and Ren et al. [96] have reviewed recent peptide immobilization methods, showing advantages and disadvantages with various single and multiple-peptide strategies. Some experimental work with peptides is very encouraging, for example that of Hoesli et al. on fibronectin-derived peptides [97] and of Hao et al. on the cyclic octa-peptide LXW7 [98] but they are still in the pre-clinical phase, with significant barriers with respect to structural stability, capturing specificity, biological functionality, and retention of endothelial cells under physiological levels of wall shear stress.

#### 3.3.1 Perspectives on endothelialization

Preconditioning of devices with endothelial cells or their precursors is neither practical nor cost-effective. Neither transanastomotic nor transmural progression of endothelial cells is effective which leaves *in situ* endothelialization as the main alternative. However, whereas with both bone – attachment and cell – adhesion processes, mechanotransduction is certainly a major factor, which can work for and against bioactivity depending on the circumstances, any biomaterial characteristic that is intended to facilitate endothelialization has to work in opposition to mechanical stresses. The interactions between devices and flowing blood are very much dependent on wall shear stresses and flow disturbances, which do not generally favor the smooth formation of a new endothelium.

### 3.4 Materials that modulate inflammation/immunomodulatory materials

Since the host response within biocompatibility phenomena encompasses innate immunity, which itself embraces inflammation [4], it is sensible to consider the potential effects of bioactive materials on inflammation and immunity in the same section. There are, however, two essentially different aspects of this, involving bioactive mechanisms that are intended to modulate the inflammatory and immune responses to biomaterial – based products and the use of immunomodulatory biomaterials as components of therapies for serious diseases, especially cancer.

#### 3.4.1 Bioactive materials for the local modulation of inflammation

As noted by Yu et al., there are some strategies aimed at modulating the local host response through passive minimization of protein adsorption [99]. Generally, these non-specific interactions have no practical value, and, anyway, could not be considered as bioactive processes. These authors go on to summarize active strategies which include the use of heparin-based coatings, Factor H-binding peptides, other peptides, receptor antagonists, carbohydrate-based systems amongst others.

Although many cell types are associated with innate immunity and inflammation, it has been the dominant macrophage that has received most attention with respect to modulation [100]. Some methods have relied on the local release of bioactive molecules, through physical entrapment or diffusion processes, but these tend to suffer from unpredictable payload release kinetics and limited bioactivity longevity. Huyer et al. have tried to overcome these difficulties through the incorporation of the active components into the backbone of carrier materials in the surface [101]; they specifically incorporated the small molecule itaconate, which has powerful immunoregulatory properties, into degradable polyesters, which provides a sustained presentation of the active molecule at the surface and allows recapitulation of the regulation of macrophage inflammation. Tan et al. have immobilized the cytokine interleukin-4 on the surface of electrospun polycaprolactone, which upregulated anti-inflammatory M2 macrophage genes [102].

**Fig. 6:**
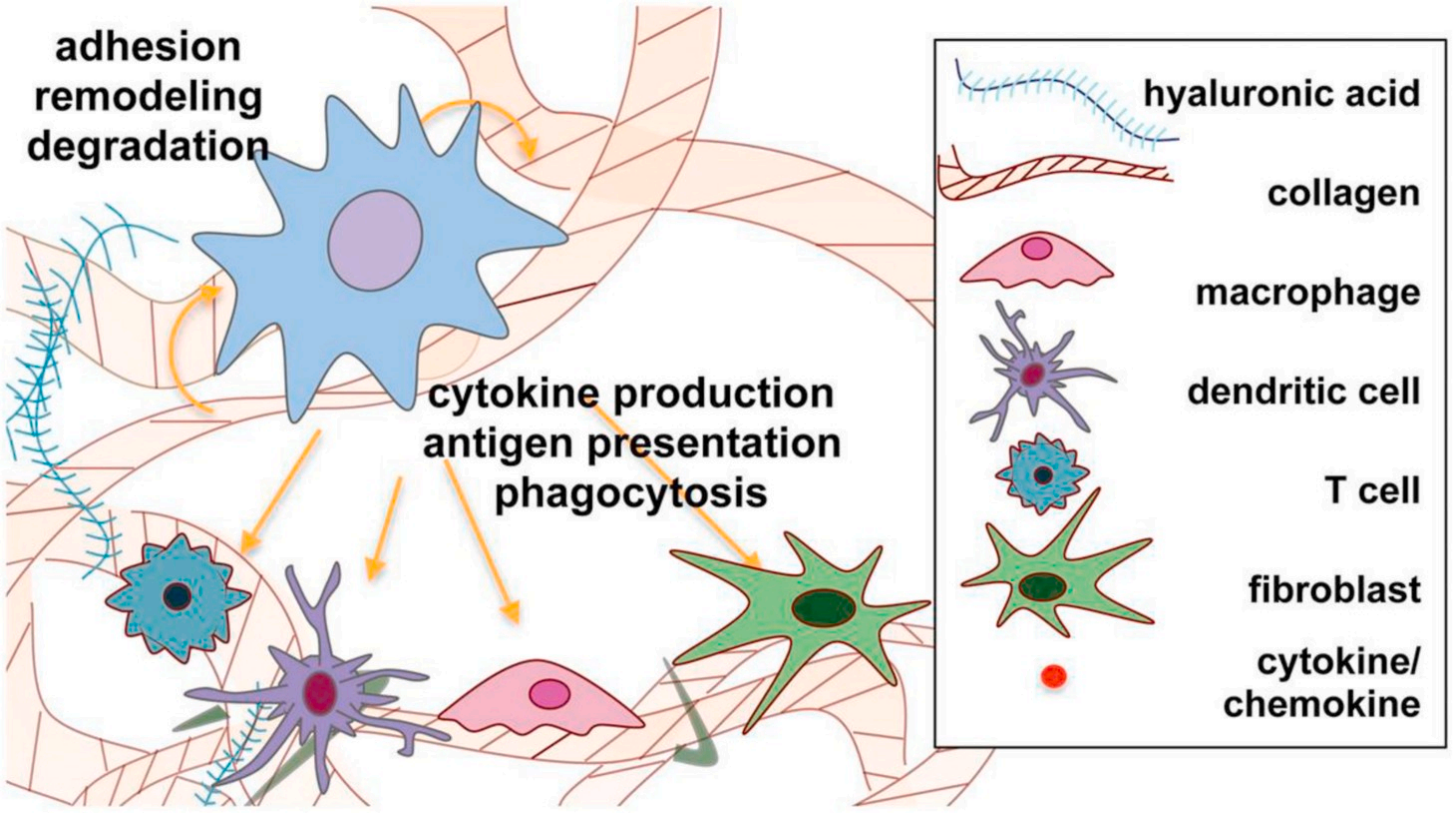
Schematic representation of dynamic cell–matrix crosstalk during immune cell–matrix interactions. Material–cell crosstalk is represented through degradation of matrix by enzymes produced by cells, the production of ECM proteins by cells, and the immunomodulatory domains in the ECM described in this review. Cellular crosstalk is depicted via the production of, and interaction with, cytokines and chemokines by the various cell types. Reproduced from Rowley et al., [103] Adv. Healthcare Mater. 8 1801578, with permission.

The challenges and prospects for immunomodulatory biomaterials have been extensively reviewed recently by Rowley et al. [103] and Liu and Segura [104]. The former specifically addressed the potential for utilizing ECM – derived materials, both those using full-length proteins such as collagen or fibrin, or hyaluronic based molecules, and those using immunomodulatory domains such as RGD, MMP-sensitive peptides or leucocyte-associated immunoglobulin-like receptor-1, as shown in Fig. 6. The latter paper focused on injectable hydrogel systems.

#### 3.4.2 Biomaterial-assisted immunomodulation for targeted therapies

As reviewed by Nakkala et al., there has been considerable progress in the design and application of immunomodulatory biomaterials for therapies with chronic inflammatory diseases [105]. The conditions that are being targeted include acute inflammatory diseases, myocardial infarction, spinal cord injury, osteoarthritis, inflammatory bowel disease and diabetic foot ulcer. These authors identify three groups of biomaterials used for immunomodulation, nanoparticles, hydrogels and scaffolds. It is emphasized here that any of the systems within these groups that operate *via* release of anti-inflammatory drugs cannot be considered as bioactive materials *per se*; the material itself, in whatever form it is presented, must have this effect to be included in the concept of bioactive materials.

There are three broad strategies for the preparation of immunomodulatory nanoparticles, those that have targeting ligands that mimic cell surface properties, those that are coated with cell membrane components and liposomes engineered with cell membrane proteins. Among the examples of such nanoparticles are peptide P12 conjugated to gold nanoparticles that can modulate Toll-like Receptor (TLR) signaling and PEG nanogels that cause a decrease in TNF-α levels in LPS-activated peripheral blood mononuclear cells [105]. Many hydrogel preparations used to regulate inflammation perform through the release of ant-inflammatory agents, but several have intrinsic anti-inflammatory properties themselves, such as certain polysaccharides and proteoglycans which could capture and scavenge pro-inflammatory factors, including IL-8, MCP-1, MIP-1α, TNF, MIP-1β and IL-1β. Scaffolds can function in several ways, typically through surface modification; for example IL-4 functionalized nanofibrous scaffolds can enhance M2 macrophage polarization as can PEO-NH_2_ stabilized gold nanorods.

One of the more promising examples of the scaffold approach involves short synthetic DNA structures [106]. The rationale for this work was stated to be the clinical need for robust conjugation strategies to functionalize the surfaces of biodegradable materials with suitable biomolecules (such as proteins and antibodies) at sufficiently high density to present multiple moieties at precisely controlled ratios. Polymer-DNA oligonucleotide amphiphilic molecules were synthesized with variable, but controlled, choice of polymer and DNA length. These provided a range of immune modulators, such as antigens, costimulatory ligands and cytokines that could be loaded onto these cell-engaging polymeric particles with intact bioactivity. These systems were primarily directed towards primary T-cell activation in cancer therapy using intratumoral injection.

Adu-Berchie and Mooney have further discussed the rationale for the design of biomaterial scaffolds as local niches for immunomodulation [107]. Depending on the precise goal, materials that either enhance the local inflammatory response or which are inherently nonimmunogenic may be considered. Many of the systems under development are multi-functional and can act both as drug delivery agents and as bioactive components. For example, the authors describe an injectable bone-marrow-like scaffold, developed to enhance T-cell regeneration and immunity after allogenic hematopoietic stem cell transplantation. This PLG-PEG based cryogel releases BMP-2 that recruits stromal cells for induction of bone formation, and also stably presents Notch ligand Delta-like ligand 4 to regulate T cell lineage commitment. This dual functionality has been further discussed by Mooney, specifically in relation to cancer therapy. Several strategies have been introduced that target M2 to M1 repolarization of tumor-associated macrophages involving molecule – delivery processes and direct stimulation, for example with ferumoxytol iron oxide or manganese dioxide nanoparticles [108]. Oakes et al. expanded on this theme in an essay on the mutual roles of biomaterial carriers and loaded immune cues in the modulation of innate immune responses [109]; of crucial importance here are those tunable biomaterial properties, such as surface charge, surface functionalization and degradation profiles that modulate the uptake of nanoparticle uptake by antigen-presenting cells. In allogeneic cell therapies for the treatment of Type I diabetes, Samojlik and Stabler have pointed to a similar combinatory approach that targets multiple activation pathways, with both cellular encapsulation to block direct recognition pathways and localized immunomodulatory agents that target indirect pathways and memory T cells [110].

#### 3.4.3 Perspectives on anti-inflammatory and immunomodulatory bioactive materials

Attempts to control implant-mediated inflammation through passive minimization of proteins have little practical value. Incorporation of anti-inflammatory molecules into biomaterials intended for subsequent release are also of limited value because of poor control of release kinetics and limited longevity; the use of such strategies means that the products are no longer bioactive devices but drug-device combinations, which has attendant safety and regulatory issues. It is possible for anti-inflammatory agents to be chemically incorporated into the biomaterials themselves, where they can exert surface modulated biological effects, especially focusing on control of macrophage phenotype, but these do not yet have much clinical utility. The better option, of course, is to use biomaterials in the products themselves that are minimally pro-inflammatory.

With respect to immunomodulatory materials that are targeted as therapies for specific disease conditions, most attention has been given to nanoparticle, hydrogel and scaffold systems; many of these are multifunctional, with both drug delivery and surface-controlled bioactivity contributions. Targeting macrophage polarization and the blocking of recognition pathways are among the relevant mechanisms.

### 3.5 Materials with antioxidant characteristics

There are many contributions to the biomaterials literature which claim to show bioactivity on the basis of anti-oxidation properties. However, there is no materials characteristic that allows such a description; a review of this literature indicates that performances of these materials are virtually always due to the release of some molecule (usually naturally occurring) that has some antioxidant character, for example rosemary extract contained within polylactic acid-based materials [111], and, as such these cannot be considered as bioactive materials and so will not be discussed further here.

### 3.6 Anti-infective biomaterials

As recently reviewed by Arciola et al., implant infections are one of the most frequent and severe complications associated with the use of biomaterials [112]. Yavari et al. have described the general mechanisms by which bacteria, especially commensal skin bacteria such as *Staphylococcus aureus*, produce biofilms on implant surfaces which protect the bacterial niche from the host defensive environment [113]. Systemic prophylactic antibiotics are often used for infection prevention [114], as are local antibiotic release processes [115], but these do not constitute bioactive material strategies. More relevant are bactericidal nanopatterns and surface biophysical cues, surface chemistry features and antimicrobial peptides [113].

#### 3.6.1 Bactericidal nanostructures

According to Linklater et al. [116], the topographical modification of a material to prevent bacterial cell adhesion or impart mechanical killing properties to the substrate, may constitute a very effective modality for controlling biomaterial-related infection. Their concept of mechano-bactericidal surface features depends on matching the dimensions of the geometric patterns of the surface with the parameters of the bacterial cell wall (especially elasticity). A width of the cap of surface features in the region of 10–100 nm seems to be preferable, with heights of features that are sufficient to prevent the bacterial membrane from contacting the substratum, while providing maximal stretching of that membrane. In effect, the bacterial attachment to a nanostructured surface is a counterbalance between the adhesion energy and the deformation energy of the bacterial membrane. Although these antibacterial mechanisms have been studied with great interest following comparisons with the apparent role of nanopatterned cicada wing surfaces with antibacterial activity [117], there is no widespread agreement on these energy-based mechanisms [118], and their translation into clinical practice is far from assured in the light of difficulties with maintaining critical nanostructural features within the aggressive biomechanical environments associated with many implant systems [115].

#### 3.6.2 Biomaterial and surface chemistry

Over several decades, experimental work has been published on the apparent antimicrobial activity of a wide variety of biomaterials with differing chemical features. These include chitosan and some other polysaccharides [119], silver [120], zinc oxide [121] and selenium [122]. It is difficult to derive any consensus about mechanisms from the collection of largely *in vitro* data that has been generated with these and other materials chemistry. Often, the material under discussion is in nanostructured form, where contributions from surface chemistry and nanotopography are impossible to separate. In addition, many of the material formulations are complex, indeed of hybrid character, and some components will be released rather than staying surface bound. It is not possible to derive bioactive pathways from this selection of materials. These difficulties have been highlighted by Chouifa et al. who attempted to review the surface modification techniques and coatings that could render titanium anti-bacterial, but which tended to highlight problems rather than current successes [123].

#### 3.6.3 Antimicrobial peptides

Antimicrobial peptides (AMPs) are peptides that possess broad spectrum antibiotic activity against most bacterial pathogens [124]. It is possible for AMPs to influence biofilm formation or to have a direct effect on bacteria, these different mechanisms determining if and how they can be incorporated into strategies to minimize implant-related infections. As discussed by Kazemzadeh-Narbat et al.*,* AMPs are evolutionarily conserved small molecules that are components of human innate immunity [125]. They disrupt the bacterial cell membrane through electrostatic interactions and lyse the cell. These interactions are based on a higher net negative charge of bacterial membranes due to the presence of acidic phospholipids in the outer layer compared to the cholesterol and phosphatidylcholine in mammalian cell membranes.

**Fig. 7:**
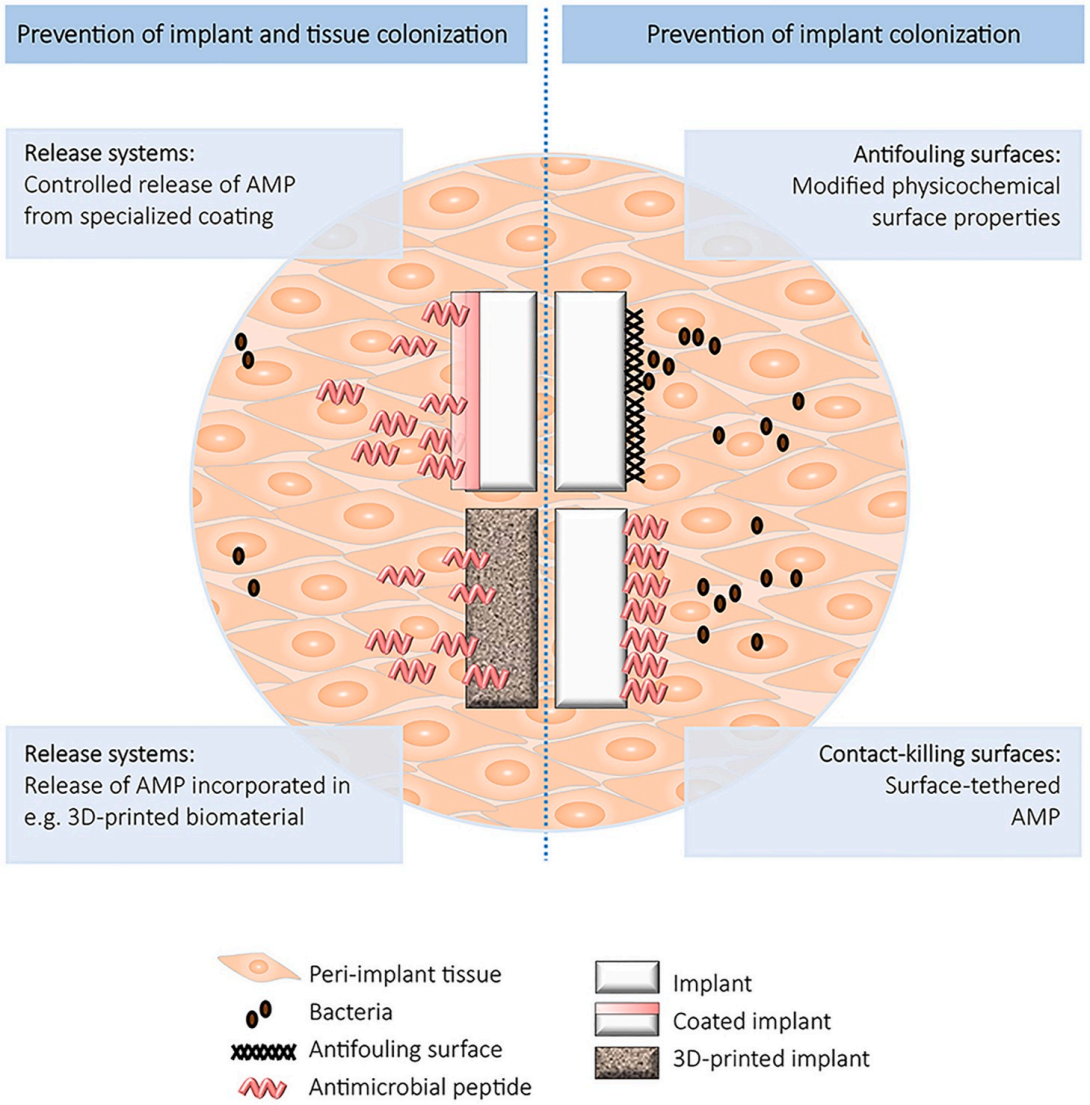
Schematic overview of strategies to prevent implant (right) and implant plus tissue colonization. Reproduced from Riool et al., [126] Front. Chem. 5 63, with permission.

There is uncertainty about how AMPs can exert protective effects against micro-organisms in practical situations since, as with other bioactivity scenarios, there is the possibility of both surface activity and controlled release phenomena, as shown in Fig. 7 [126]. Surface modification of biomaterials by chemical or physiochemical methods can result in effective antimicrobial activity *in vitro* but there are limited effects in tissues surrounding implants. Coating strategies on biomaterials include polymer brushes and dopamine-based layers. The former constitutes an assembly of high-density polymer chains that can be tethered chemically to a surface at one end, where dangling chains repel each other and stretch lengthwise, providing a flexible linker between the substrate and the AMP [127]. It is important to note that polymer brushes by themselves can reduce adhesion of bacteria without killing them. Polyethylene oxide can decrease the adhesion of *Staphylococci* and *Escherichia coli* but not of the more hydrophobic *Pseudomonas aeruginosa*. When antimicrobial agents such as peptides are immobilized on the polymer brushes, the spacer length and accessibility of the peptide are important. Ideally the polymer brush – peptide layer provides enough conformational freedom for the peptide to interact with the bacterial membrane, while preserving the peptide activity after immobilization. Important factors are the tethering point of the peptide to the polymer and the extent and distribution of the hydrophobic and charged residues within the peptide sequence. The second strategy referred to above involves the mussel-inspired adhesive hydrogel, self-polymerizing dopamine, which can be deposited on several types of biomaterial surfaces, including metals and polymers, and to which antimicrobial peptides can be tethered; these may have both anti-biofilm and antimicrobial properties [128].

There have been several attempts to improve antimicrobial peptide performance in infection control in the last year or so, including hierarchical architecture of peptide-conjugated polymer brushes [129], molecular engineering and self-assembly [130], truncated antimicrobial peptide sequences that are combined with protein films [131] and a combination of genetic algorithms and machine learning for peptide design [132]. It remains to be seen whether these result in clinical utility.

#### 3.6.4 Perspectives on infection control bioactive materials

One of the most clinically relevant aspects of biomaterial-related infection control is that of catheter-associated infections. As reviewed by Ricado et al.*,* despite several decades of intensive research and development involving those strategies discussed in the previous sections, and others, efficacy data obtained from *in vitro* studies is not properly reflected in the clinical setting [133].

### 3.7 Anti-thrombogenic materials

In the healthy individual, a stable blood circulation implies that there is a supply of oxygen and nutrients to all tissues and the removal of metabolites from them. Part of the physiological system that ensures this stability is the finely tuned balance between pro- and anti-coagulant factors in blood and blood-contacting tissues. This balance may be disturbed in some disease states and also when implanted or invasive medical devices contact the blood, leading to either bleeding when anti-coagulant factors dominate and thrombosis or infarction when pro-coagulant factors are in control. Biomaterial surfaces themselves are unable to provide anti-coagulant properties and, in contrast, may induce pro-coagulant activity. In many clinical situations, the latter possibility is countered by the use of systemic anticoagulant drugs, but finding the right balance over time is not trivial and there are risks of triggering bleeding episodes.

As discussed by Maitz et al., many types of strategy have been designed for the presentation of biomaterials with suitable hemocompatibility characteristics through modification of their surfaces [134]. These strategies may be conveniently discussed in terms of passive and bioactive behavior. Passive approaches, which for obvious reasons will not be considered further in his essay on bioactivity, could include methods to suppress protein adsorption through deposition of substances that rely on either surface charge or hydrophobicity for their effects. Bioactive surface modifications tend to be based on substances that mimic the normal blood vessel wall. The specific case of endothelialization has already been addressed in section C above. Other strategies involve the use of anticoagulant or fibrinolytic molecules, and synthetic or biological inhibitors of surface reactions. Effects may be direct or indirect, and agents may be released or immobilized [134].

#### 3.7.1 Immobilized anticoagulants

Many surface modification techniques have been developed with the intention of minimizing the risk of thrombus formation [135]. Most of these rely on increasing hydrophilicity in order to limit protein adsorption with the lower surface energy; these include polyethylene oxide/polyethylene glycol and phosphorylcholine; although *in vitro* experiments can show some beneficial effects of this passive approach, these have rarely been translated into clinical success.

Far fewer attempts have been made to develop bioactive antithrombogenic surfaces. Some of these have involved nitric acid (N0) producing coatings; NO is present in native endothelium, where it influences several important pathways of cardiovascular homeostasis. With respect to biomaterials surfaces, modification strategies have focused on mimicking these functions by the use of NO-generating coatings [136]. For example, the non-toxic NO donor S-nitroso-N-acetylpenicillamine can be impregnated into polymer surfaces for controlled release over a few weeks [137].

The majority of effort in this area has been devoted to heparin coatings [138]. Heparin is a naturally occurring linear polysaccharide that has been used clinically as an injectable anticoagulant for decades [139]; for much of that time it has been considered as a coating on blood-contacting surfaces of medical devices. Heparin produces its major anticoagulant effect by inactivating thrombin and factor Xa *via* an anti-thrombin - dependent mechanism. It binds to antithrombin (AT) by means of a high-affinity pentasaccharide, which is present in about one-third of heparin molecules. Heparin must bind to both the coagulation enzyme and AT in order to inhibit thrombin. There are two approaches to the heparinization of biomaterials surfaces, the first involving eluting technologies for heparin release, which cannot be considered as bioactive strategies since they rely on drug release mechanisms, while the second uses permanent covalent immobilization. This latter immobilization concept relies on the strong net negative charge of the molecule, the abundance of carboxyl groups and the presence of several active functional groups, for effective attachment. The mechanisms whereby immobilized heparin exert anticoagulation behavior are similar to those for systemic heparin preparations, although reaction kinetics are very different. *In vitro* models show that surface bound heparin reduce the formation of activated thrombin, reduce complement activation and reduce both platelet adherence and activation. It should be noted that there are no ‘new bioactivity pathways’ here, the heparin functioning according to its classical properties; it is also noteworthy that the functionality of the immobilized heparin is dependent on its conformation once attached to the surface, and that the retention of this functionality is problematic in view of *in vivo* degradation of the heparin, especially under flow conditions. Most clinical applications of these immobilization technologies are of a short-term nature. Attempts have been made to improve performance through the development of heparin-mimicking polymers [140] and other bioengineered heparins and heparan sulfates [141].

#### 3.7.2 Other immobilized molecules

Attempts have been made to modify biomaterials surfaces with other agents that can interfere with the clotting cascade, including the promotion of thrombolysis/fibrinolysis, using, for example, urokinase [142] or tissue plasminogen activator [143]. While promising *in vitro* results may be obtained, once again clinical applications are difficult to demonstrate and some of these technologies, especially those which reply on molecule release, may have adverse effects [134].

#### 3.7.3 Perspectives on control of thrombogenicity by bioactive materials

Some success has been achieved with surface coatings in short-term blood-contacting situations. The majority are dependent on the release of an active component rather than surface bioactivity itself; these systems rely on continued release under physiological conditions and the avoidance of both diminished diffusion through deposition of plasma proteins and inactivation through enzyme- or other molecule degradation or denaturation processes. Long-term anticoagulation by the sole use of surface modifications has proven difficult to achieve.

### 3.8 Materials that promote wound healing

There are three potential areas of interest with respect to wound healing. One concerns tissue engineering/regenerative medicine approaches to tissue loss, which is outside the scope of this essay. The second related to the use of biomaterials, in the form of dressings, that either enhance the healing of traumatic or surgically acquired breach of tissue continuity or, at least protect the area while natural healing takes place, while the third concerns the attempt to modify wound healing in association with the use of implantable devices.

Central to the second and third uses is the fact that evolution has determined that most tissues, especially skin, have powerful mechanisms of healing, or natural wound closure, even if in many situations the repair is by fibrous scar tissue. The main reasons why dressings are used are to assist in the mechanical apposition of wound edges, to assist in the prevention of wound infection and to provide the optimal environment, primarily the fluid environment, in which the natural healing process takes place. Rarely is it required to accelerate, or promote, wound healing in otherwise healthy individuals. Traditional wound dressings have been passive non-occlusive natural or synthetic fiber based substances but the need to maintain a hydrated environment has led to the use of semi-permeable materials such as hydrocolloids, hydrogels and foams. Many currently used dressings incorporate agents with pharmacological activity and some use natural biopolymers such as chitosan [144], keratin [145], silk [146], alginates [147] and hybrids or composites that may marginally assist in the healing process. Some are compounded with growth actors or antimicrobial agents in attempts to produce better clinical performance [148].

With respect to implantable devices, there are several situations where the progress of wound healing is absolutely critical for good clinical outcomes, the most obvious example being that of hernia repair. Hernias are associated with tissue breakdown, where internal organs penetrate through the wall of muscle or other tissue that normally contain it, the resolution of which normally involves using an implantable mesh in the area of protrusion, with the objectives of constraining the protruded tissue and encouraging healing of the defect. Most hernia repairs are successful, but failure to achieve a satisfactory outcome may arise from infection of the wound area, failure of wound healing giving rise to dehiscence, adhesions to other tissue or organs and some subjective features such as chronic pain [149]. Most hernia meshes are made of polypropylene, with a few using polyester. In view of the numbers of procedure failures (usually a small percentage of procedure, but the population is large), manufacturers of meshes have tried many ways to improve the healing process, including reduction of adhesions and lower levels of pain. Apart from variations in mesh architecture, the most common approach has involved surface modification of the mesh with some so-called bioactive material, intended to improve the biological outcome of the healing process. Examples have included coatings of oxidized regenerated cellulose [150], omega-3 fatty acid gel [151], hyaluronic acid [152] and collagen [153]. In most cases there was little scientific rationale for the modifications, and, with a few exceptions, the clinical outcomes were poorer than unmodified meshes.

It remains the case that bioactive surfaces are usually ineffective in promoting wound healing.

## 4 Conclusions and opinions

Several generic points arise from this analysis of bioactivity.

The first concerns the difference between inert and active biomaterials. As noted in section II, no material can be both inherently inert and intrinsically active at the same time. The titanium and PEEK materials I mentioned are inert in the physiological environment. If changes are made to those materials that give some ‘activity’, they are no longer either titanium or PEEK.

If a material exerts biological activity solely by the release of pharmacologically active agents, from a scientific perspective they cannot be considered as bioactive materials and the products will almost certainly be designated as biomaterial – drug combinations by regulatory authorities. It is possible that under some circumstances, a biomaterial that incorporates a drug may display pharmacological/biological activity by a combination of surface-controlled mechanisms and the effects of drug release. Each of these combinations will have to be considered (both from regulatory and scientific perspectives) on their own merits.

Bioactivity of biomaterials, therefore, involves the modulation of biological activity by virtue of the characteristics of the material surface, or more specifically by the characteristics of the interfacial region that incorporates the material surface and the immediate local host tissue. Just as biocompatibility cannot be considered solely as a property of a material but rather of a biomaterial – host system [4], so bioactivity cannot be considered solely as a property of a material but rather of the biomaterial surface – local host system.

**Fig. 8:**
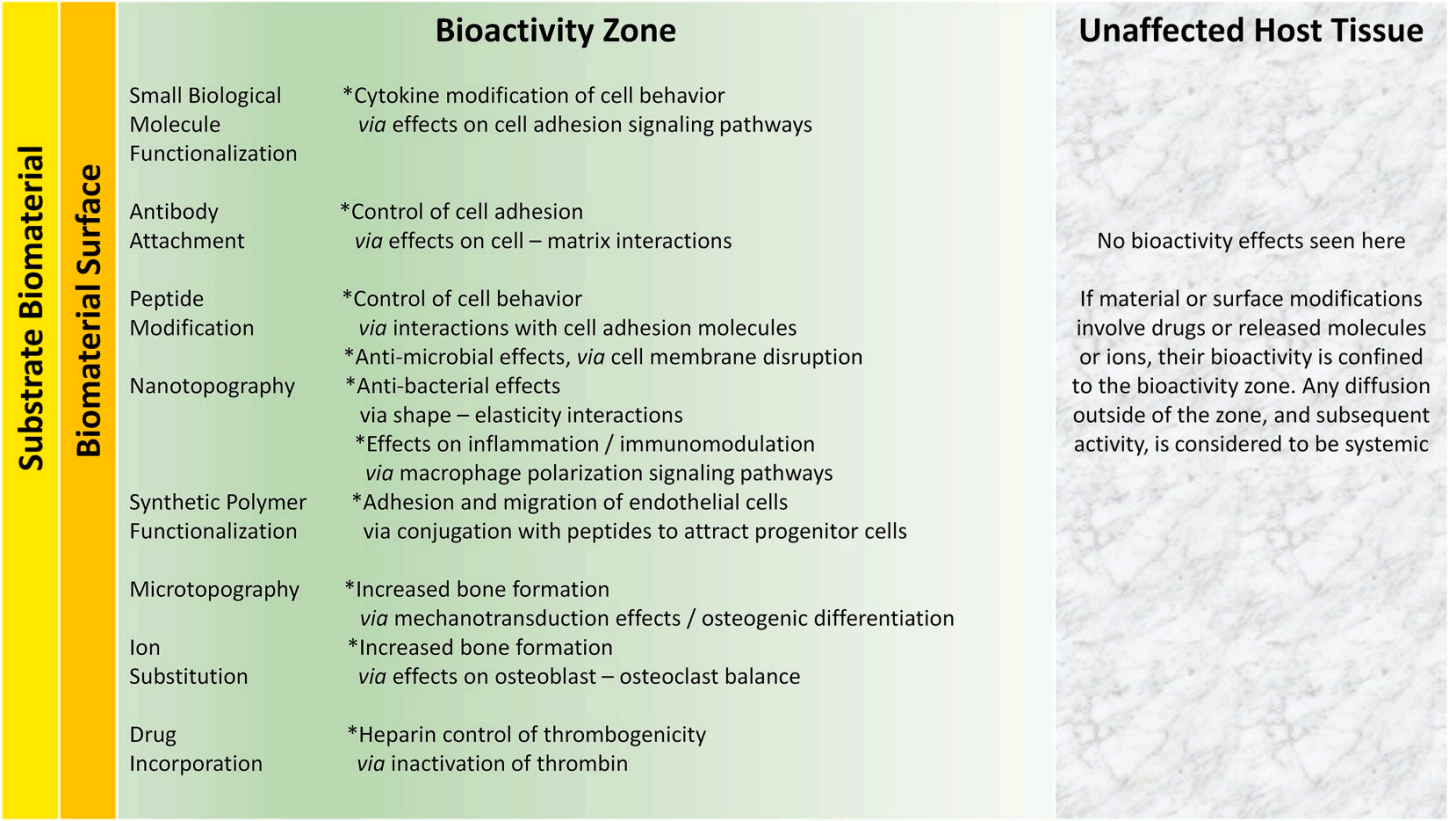
Representation of The Bioactivity Zone. The surface of a biomaterial may be presented with small biological molecule functionalization, nano- or micro-topography, antibody or peptide attachment, synthetic polymer functionalization, ion substitution in the surface layer or the bulk material, drug incorporation by surface attachment or within the material, or any other relevant feature. The effects of this surface presentation may be confined to the immediate parts of the bioactivity zone or may be seen within other parts of this zone, extending towards the normal host tissue. Effects of any released moiety outside the bioactivity zone are considered to be systemic. The width of the bioactivity zone may be of single-cell dimensions or greater than a millimeter. Observed phenomena within the zone include, but are not limited to, effects of cell adhesion signaling pathways and inflammation/immunomodulation signaling pathways, the regulation of osteoblast/osteoclast balance through effects on various bone regulation pathways, effects on bacterial cell membranes, topographically-induced mechanotransduction effects, active interference with blood clotting mechanisms and on endothelialization processes.

Although the term ‘bioactive material’ is widely used and, through common usage, has a well understood general meaning (as evidenced by the contents of this journal), from a scientific perspective, it would be useful to concentrate on this interfacial region, which could be considered as ‘*the bioactivity zone’*. This concept is expressed graphically in Fig. 8.

In section II, I noted that common sense might suggest that an entirely non-biological change to a material surface could not be considered as a part of a bioactivity phenomenon. Experience now shows that this is not the case, since surface modifications that impart specific topographical features, or which alter micromechanical properties, can influence biological activity in this bioactivity zone.

Just as a very detailed analysis of clinically – relevant biocompatibility phenomena [4] indicated that they are of two generic types, involving either mechanotransduction effects or sterile inflammation associated with the chemical characteristics of the material, so the current analysis of bioactivity phenomena shows that these are either primarily due to topographical and/or micromechanical characteristics, or to biologically active species that are presented, or otherwise made available, in the bioactivity zone.

Examples of topographical/micromechanical contributions to bioactivity are seen with modulation of the osteoblast – osteoclast balance, and osteogenic differentiation within the bioactivity zone in bone contacting devices, the nanotopographical regulation of cell adhesion in soft tissue and the properties of bactericidal nanostructures.

Examples of the regulation of bioactivity by biologically active species include the potential influence, especially of metal ions, on signaling pathways in bone formation in this zone, the role of cell adhesion molecules, particularly with respect to bioactive peptides in cell attachment, the alteration of macrophage polarization by the presentation of immunoregulatory molecules, the immunomodulatory effects of nanoparticles and short synthetic DNA structures, and antimicrobial peptides.

It is clear that while a great deal of experimental data exists to demonstrate the potential of such bioactivity phenomena, there are still considerable barriers to their effective clinical translation. In some situations, work over several decades has produced convincing evidence of effects within *in vitro* or simple *in vivo* models, clinical applications are still very limited in view of rapid loss of activity or other factors; heparin modification for control of thrombogenicity is a good example.

This essay has shown that there is solid scientific evidence of the existence of bioactivity mechanisms that are associated with some types of biomaterials, especially when the material is modified in a manner designed to specifically induce that activity. However, observations of simple changes in performance under *in vitro* conditions, without reference to plausible underlying mechanisms, do not contribute to that evidence.

## Funding

This research did not receive any specific grant from funding agencies in the public, commercial or not-for-profit sectors. The author has no conflicts of interest to declare.
